# Supplementary material for: Statistical and Evolutionary Analysis of Sequenced DNA from Breast Cancer FFPE Specimens
Source: bioRxiv. 2025 Oct 5:2025.10.04.680485. Preprint. [Version 1] doi: 10.1101/2025.10.04.680485 (PMC12622042; doi:10.1101/2025.10.04.680485)
Supplement: 1 [file NIHPP2025.10.04.680485V1-supplement-1.pdf]

bioRxiv preprint doi: https://doi.org/10.1101/2025.10.04.680485; this version posted October 5, 2025. The copyright holder for this preprint (which was not certified by peer review) is the author/funder, who has granted bioRxiv a license to display the preprint in perpetuity. It is made available under aCC-BY 4.0 International license.

A Appendix: Data acquisition and cleaning

A.1 Additional files

Supplementary Table 1: Sequencing statistics for all BRCA samples (file: Supplementary_Table_1.xlsx; 18 KB)

A.2 DNA sequencing quality control

Shown below are the exon coverage statistics for all samples in the cohort (Figure S1). The sequencing statistics are presented in Supplementary Table 1. They list the read statistics for all samples, subdivided into primary tumor (P1), cancerous lymph node (L1) and benign lymph node (C), used as control for the detection of somatic mutations.

The read statistics for our BRCA group are shown in Figure S2. The graph showing sequencing depth (coverage) is included as Figure S3.

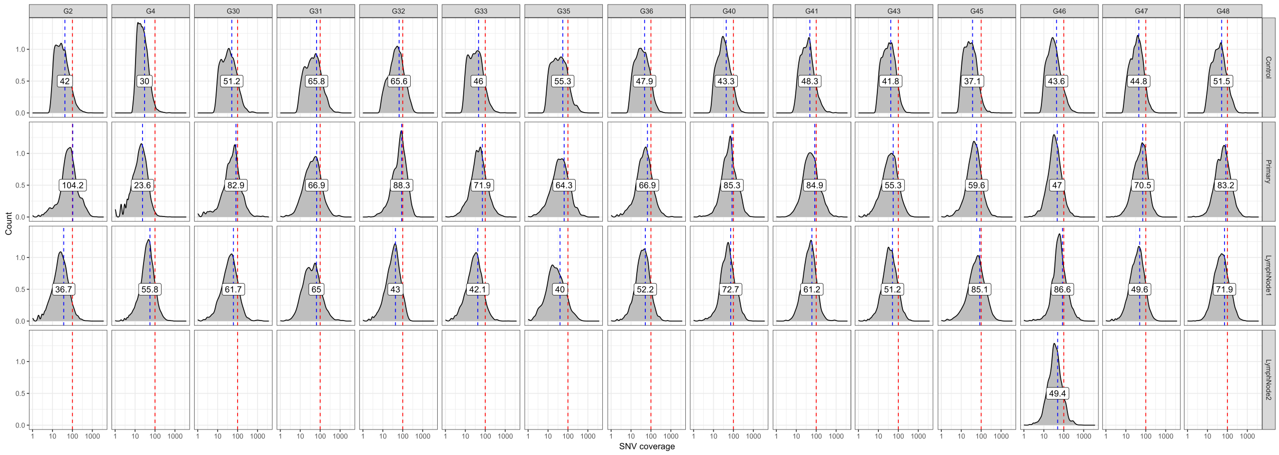

Figure S1: Exon coverage characteristics for all BRCA samples.

bioRxiv preprint doi: https://doi.org/10.1101/2025.10.04.680485; this version posted October 5, 2025. The copyright holder for this preprint (which was not certified by peer review) is the author/funder, who has granted bioRxiv a license to display the preprint in perpetuity. It is made available under aCC-BY 4.0 International license.

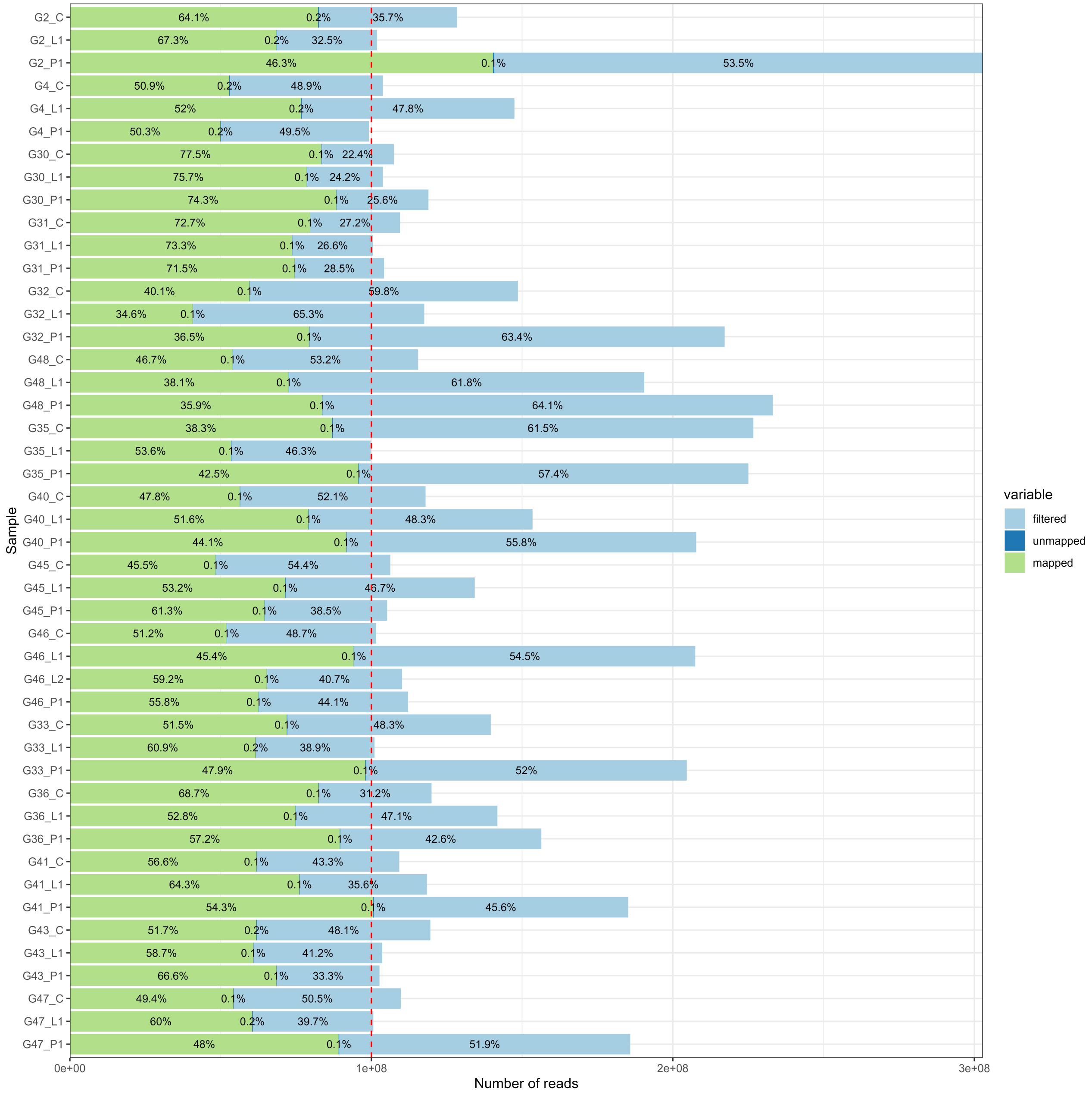

Figure S2: Read statistics for all BRCA samples.

bioRxiv preprint doi: https://doi.org/10.1101/2025.10.04.680485; this version posted October 5, 2025. The copyright holder for this preprint (which was not certified by peer review) is the author/funder, who has granted bioRxiv a license to display the preprint in perpetuity. It is made available under aCC-BY 4.0 International license.

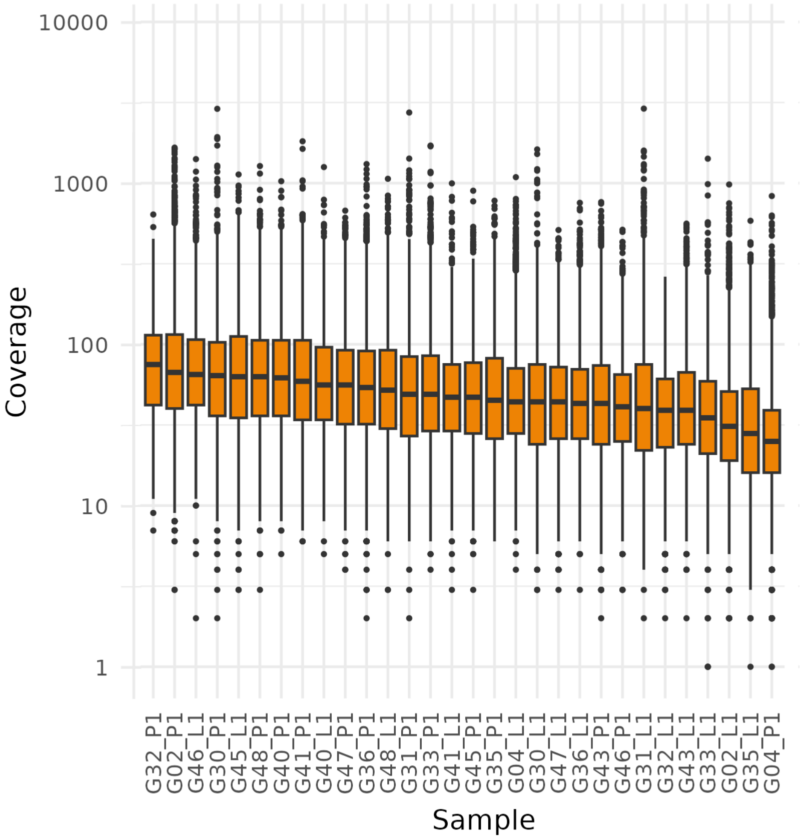

Figure S3: Sequencing coverage of SNVs and Indels across samples in BRCA cohort.

Figure S4 shows the count of variants retained in each sample after filtration with each tool and, additionally, the count of variants in the case when FPPE score threshold was changed to 0.25 in the result of DEEPOMICS. The numbers were highest in case of SOBDetector, significantly exceeding these observed after filtration with other tools and also the average number of variants observed in TCGA samples. In turn, the counts of variants obtained with other tools were too low for some of the analyses we conducted, and therefore we decided to alter the FFPE score threshold in DEEPOMICS to enrich the outcome with variants with lower allele frequency, but retaining sufficient efficacy of filtration (see Figure 5).

bioRxiv preprint doi: https://doi.org/10.1101/2025.10.04.680485; this version posted October 5, 2025. The copyright holder for this preprint (which was not certified by peer review) is the author/funder, who has granted bioRxiv a license to display the preprint in perpetuity. It is made available under aCC-BY 4.0 International license.

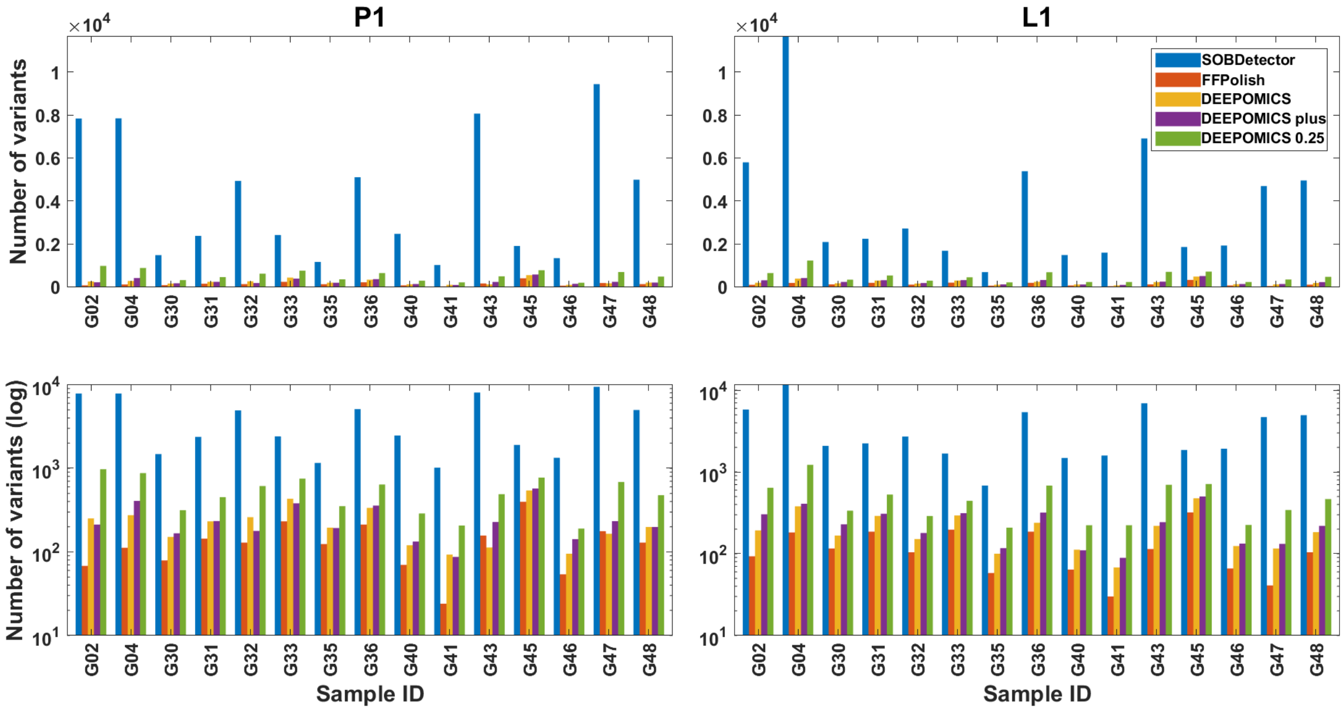

Figure S4: Count of single nucleotide variants retained in primary tumor (P1, left column) and lymph node metastasis (L1, right column) samples by each of the tools and number of variants for DEEPOMICS with adjusted FFPE score threshold (DEEPOMICS 0.25). Both sample groups are shown in linear (top row) and log (bottom row) scale.

B Appendix: Additional details of genomic analysis

B.1 Driver ploidy

Figure S5 shows the result of analysis of copy number change in genomic regions encoding driver mutations in all samples before (top panel) and after filtration with DEEPOMICS with adjusted FFPE score threshold (bottom panel). The background dashes (horizontal for P1 and vertical for L1) represent relative copy number in region with respect to assumed normal ploidy of sample equal to weighted average of all genomic regions in this sample. The plot area of both figures is divided into three major groups: tumor suppressor genes (TSG), oncogenes (OG) and a class of genes (OG/TSG) acting in different ways depending on specific context, such as e.g. a BRCA subtype or a specific mutation site.

For such estimated baseline ploidy, which in most cases is higher than 2, the proportion of copy

bioRxiv preprint doi: https://doi.org/10.1101/2025.10.04.680485; this version posted October 5, 2025. The copyright holder for this preprint (which was not certified by peer review) is the author/funder, who has granted bioRxiv a license to display the preprint in perpetuity. It is made available under aCC-BY 4.0 International license.

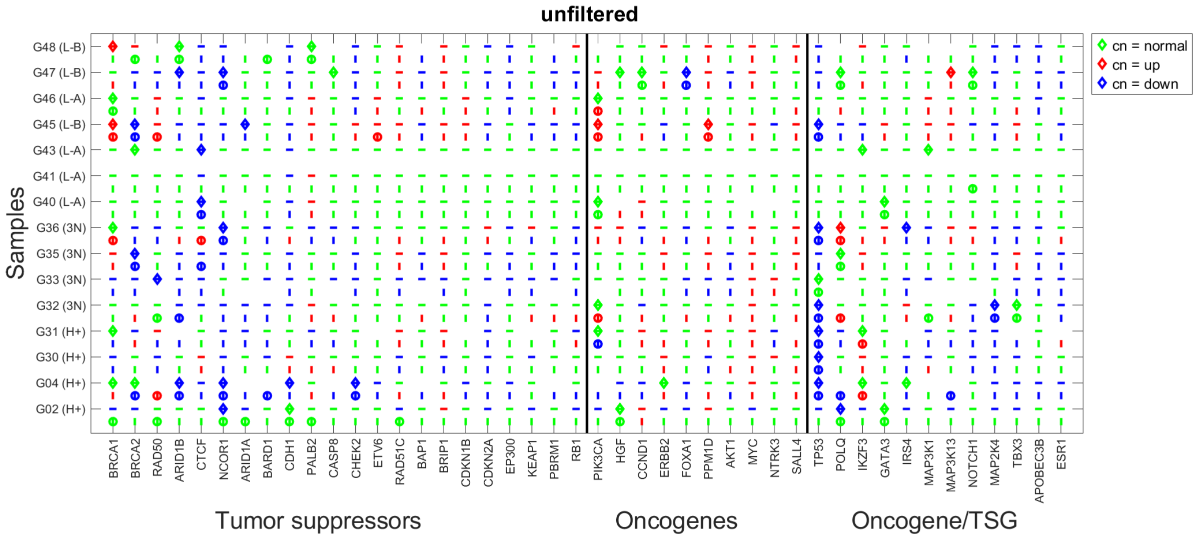

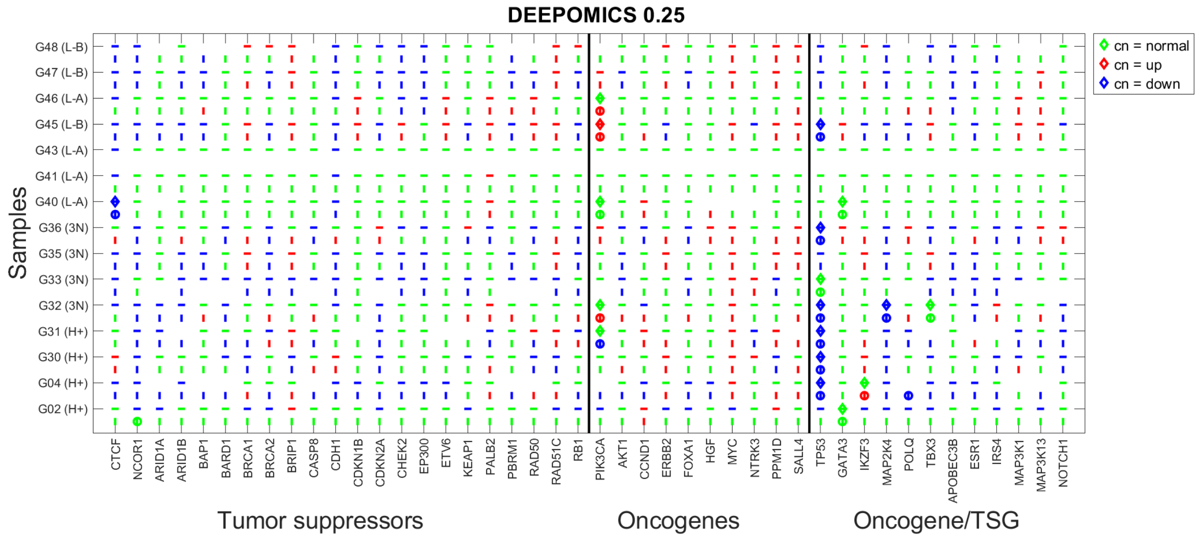

Figure S5: Ploidy in genomic regions encoding driver mutations in all P1 and L1 samples before (top panel) and after filtration with DEEPOMICS with adjusted FFPE score threshold (bottom panel). Dashes (horizontal for P1 and vertical for L1) represent relative ploidy in region with respect to assumed normal ploidy of the sample equal to weighted average of all genomic regions in this sample. The change in ploidy is encoded with color: green - normal ploidy, red - increased ploidy, blue - decreased ploidy. Additionally mutations in driver genes are marked by diamond (P1) and circle (L1). Horizontal axis of the graph is sorted by driver gene function and divided into 3 major groups: TSG's, OG's and OG/TSG's. In each of the major groups driver genes are sorted in descending number of mutational hits.

bioRxiv preprint doi: https://doi.org/10.1101/2025.10.04.680485; this version posted October 5, 2025. The copyright holder for this preprint (which was not certified by peer review) is the author/funder, who has granted bioRxiv a license to display the preprint in perpetuity. It is made available under aCC-BY 4.0 International license.

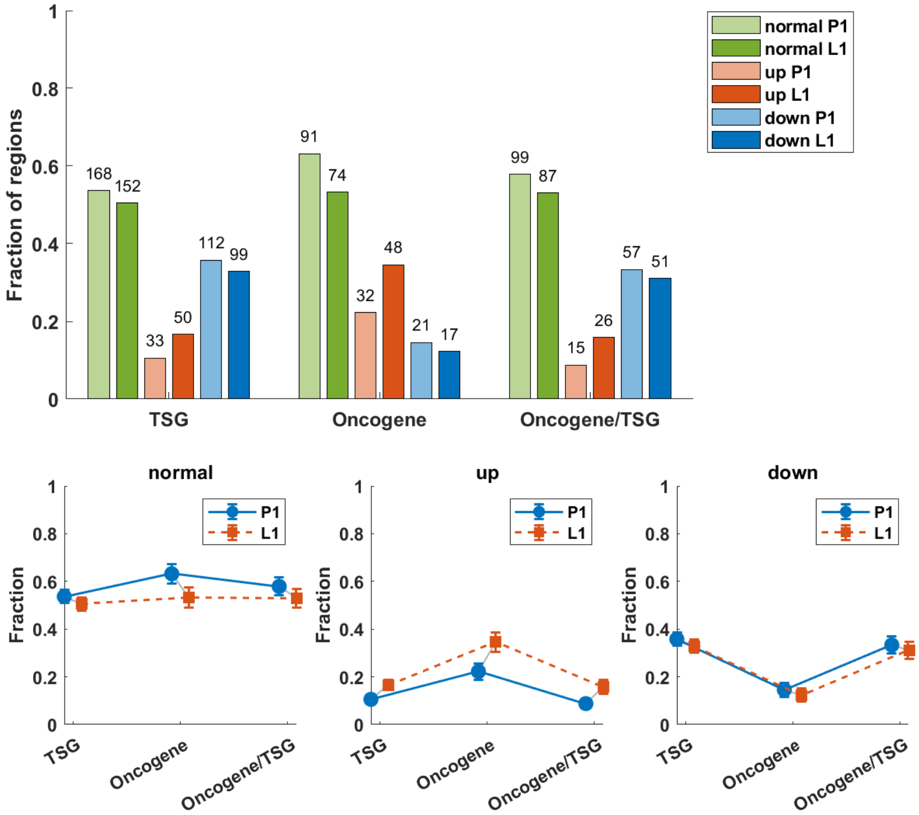

Figure S6: Statistics of copy number status in regions encoding driver mutations across all P1 and L1 samples, assuming normal ploidy of the sample equal to weighted average of all genomic regions in this sample. **Top panel:** Average copy number in regions encoding three major groups of driver genes: TSG, OG, and OG/TSG. **Middle panel:** Fractions of regions with copy number increased (red), decreased (blue) or not changed (green) with respect to assumed normal ploidy equal to 2. The height of the bar depicts proportional share in given major group, while the numbers above are the counts of regions of given type. **Bottom panel:** Fractions of regions encoding driver mutations in P1 and L1 with copy number not changed (normal), increased (up) and decreased (down) in the three major groups of driver genes. Distribution of TSG and OG in P1, comparing copy number down vs. normal vs. up (counts: down — TSG 112, OG 21; normal — TSG 168, OG 91; up — TSG 33, OG 32): $\chi^2 = 26.27$, $p < 0.001$. In L1 (counts: down — TSG 99, OG 17; normal — TSG 152, OG 74; up — TSG 50, OG 48): $\chi^2 = 29.25$, $p < 0.001$. Both results are significant at $p < 0.05$ — TSGs are enriched with copy number loss and OGs are enriched among amplifications. Total counts of regions with copy number down/normal/up (TSG, OG, OG/TSG combined) across P1 and L1 (counts: down — P1 190, L1 167; normal — P1 358, L1 313; up — P1 80, L1 124): $\chi^2 = 13.53$, $p < 0.001$. The result is significant at $p < 0.05$ — P1 shows relatively more copy number losses while L1 shows relatively more amplifications.

bioRxiv preprint doi: https://doi.org/10.1101/2025.10.04.680485; this version posted October 5, 2025. The copyright holder for this preprint (which was not certified by peer review) is the author/funder, who has granted bioRxiv a license to display the preprint in perpetuity. It is made available under aCC-BY 4.0 International license.

number gains (marked with red color in the graph) is lower than for reference ploidy equal to 2. Instead, we observe the decrease of copy number in regions containing tumor suppressor genes. The region containing TP53 gene (here classified as a mixed-role gene) is characterized by lower than baseline copy number and this effect is shared by most of the samples which have an additional mutational hit in this gene.

We checked how many BRCA-driver containing genomic regions fall in the category of increased, decreased and normal (unchanged) copy number. Results are subdivided into three groups: TSG, OG and OG/TSG. In all three groups fraction of regions with unchanged copy number is higher than in the remaining categories.

The fraction of regions with increased and decreased copy number is similar between TSG and OG/TSG groups while in the OG group, the highest increase in copy number is observed. In addition, the fraction of genomic regions with decreased copy number is the smallest.

This trend, which is most clearly shown by Figure 11 remains present also in the case of estimated baseline ploidy of the samples. All 3×2 comparisons were tested using two-tailed χ^2 test for contingency tables of gene class (TSG vs OG) or sample (P1 vs L1) versus outcome (down vs normal vs up). Counts are the numbers of genomic regions.

B.2 Site frequency spectra

In this section, we first present aggregates of all samples. In the aggregate spectra as depicted in Figure S7, shown are also the positions of BRCA drivers in the frequency spectrum. The counts of driver genes at particular VAF frequencies are marked with red and shown in the logarithmic scale.

To take into account purity and ploidy which vary across samples we calculated Cancer Cell Fraction (see Methods, Section 2.2.2) and presented it in the same form as the aggregated VAF-based site frequency spectrum (Figure S8). As in the previous case, the bars at frequencies where driver mutations were found are marked with red.

In both cases, in unfiltered and SOBDetector processed datasets driver mutations are present in almost all lowest frequency-bars (above the 0.05 threshold for VAF imposed on mutations in regions containing driver genes), while in a growing and mutationg tumor they are expected to be shared by a larger proportion of variant calls. For the FFPolish- and DEEPOMICS-filtered data most drivers fall into frequency thresholds from 0.2 to 0.5, which after correction for purity and ploidy and calculation of CCF spectrum shifts them towards frequency close to 1.0. Please see the cluster of drivers around value 1 in panels depicting CCF spectra under FFPolish and DEEPOMICS

bioRxiv preprint doi: https://doi.org/10.1101/2025.10.04.680485; this version posted October 5, 2025. The copyright holder for this preprint (which was not certified by peer review) is the author/funder, who has granted bioRxiv a license to display the preprint in perpetuity. It is made available under aCC-BY 4.0 International license.

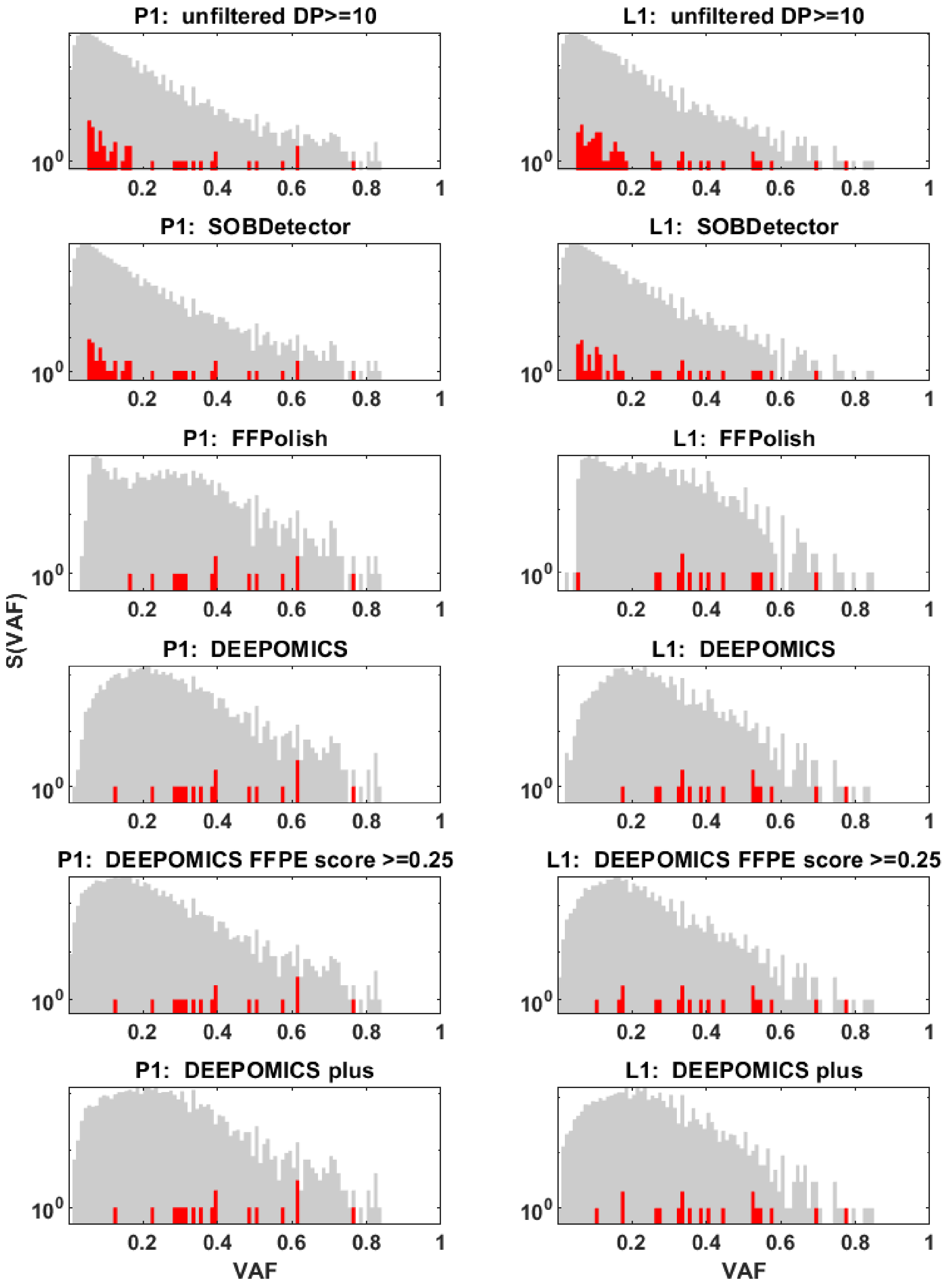

Figure S7: Site frequency spectrum (SFS) based on aggregated variant allele frequency (VAF) from patients samples from primary tumor (P1, left column) and lymph node metastasis (L1, right column) in the log scale. With red marked are bars for VAF thresholds in which mutation variants in driver genes were observed with actual number of drivers belonging to this threshold. Each panel from top to bottom was generated using different filtration method. Notice the differences on Y axis between methods.

bioRxiv preprint doi: https://doi.org/10.1101/2025.10.04.680485; this version posted October 5, 2025. The copyright holder for this preprint (which was not certified by peer review) is the author/funder, who has granted bioRxiv a license to display the preprint in perpetuity. It is made available under aCC-BY 4.0 International license.

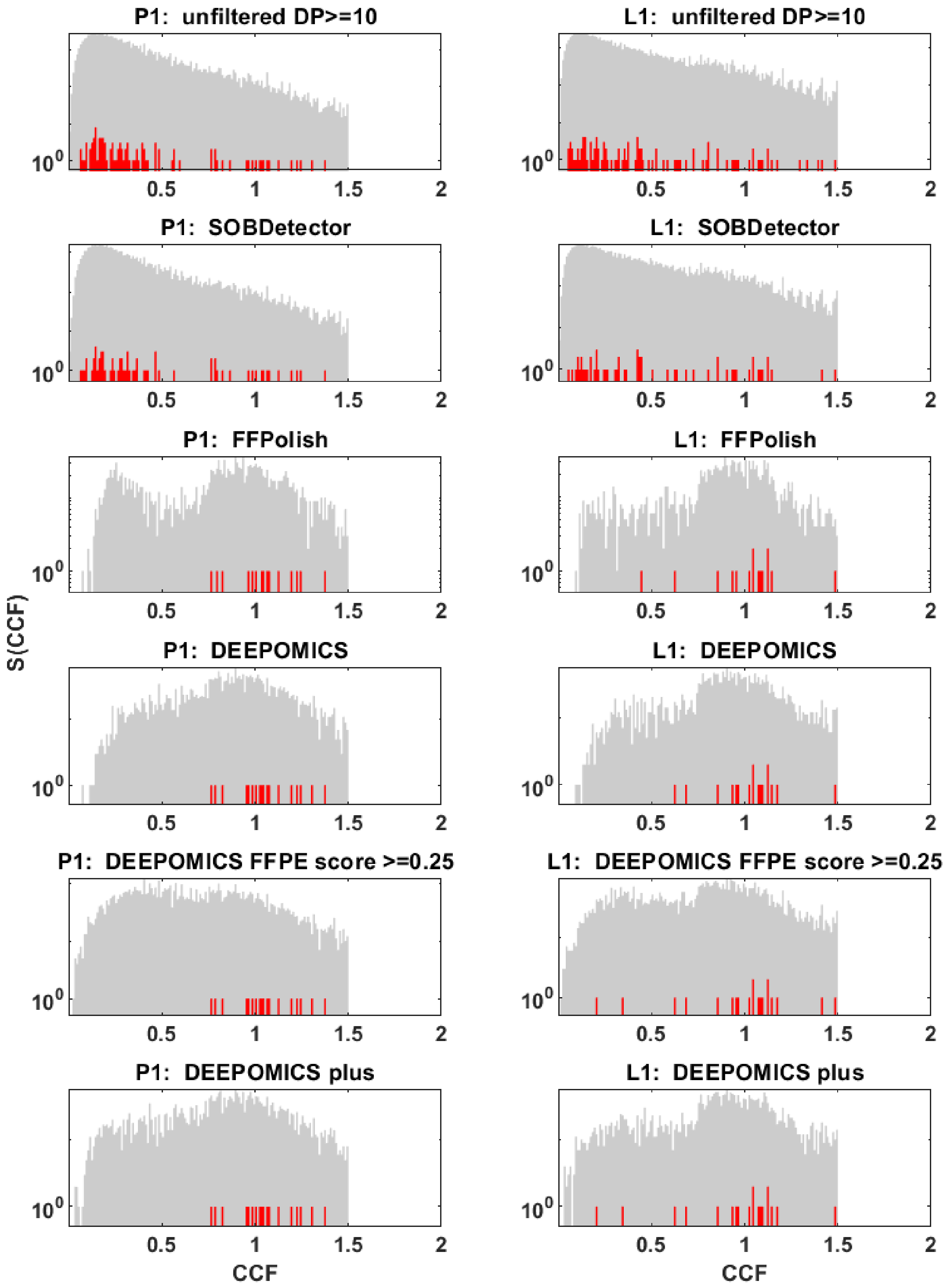

Figure S8: Site frequency spectrum (SFS) based on aggregated cancer cell fraction (CCF) from patients samples from primary tumor (P1, left column) and lymph node metastasis (L1, right column) in the log scale. With red marked are bars for VAF thresholds in which mutation variants in driver genes were observed with actual number of drivers belonging to this threshold. Each panel from top to bottom was generated using different filtration method. Notice the differences on Y axis between methods and the range of X scale (CCF not rescaled).

bioRxiv preprint doi: https://doi.org/10.1101/2025.10.04.680485; this version posted October 5, 2025. The copyright holder for this preprint (which was not certified by peer review) is the author/funder, who has granted bioRxiv a license to display the preprint in perpetuity. It is made available under aCC-BY 4.0 International license.

filtrations. In additon, see Equ. (7), which explains the relationship between the scales of the CCF- vs. VAF-based spectra.

B.2.1 Listing of SFS and CCF spectra of all specimens

Figures S9-S12 represent site frequency spectra for individual samples, based on VAF (Figs S9 and S10) and CCF (Figs S11 and S12). Shown are results before (Figs S9 and S11) and after filtration (Figs S10 and S12).

bioRxiv preprint doi: https://doi.org/10.1101/2025.10.04.680485; this version posted October 5, 2025. The copyright holder for this preprint (which was not certified by peer review) is the author/funder, who has granted bioRxiv a license to display the preprint in perpetuity. It is made available under aCC-BY 4.0 International license.

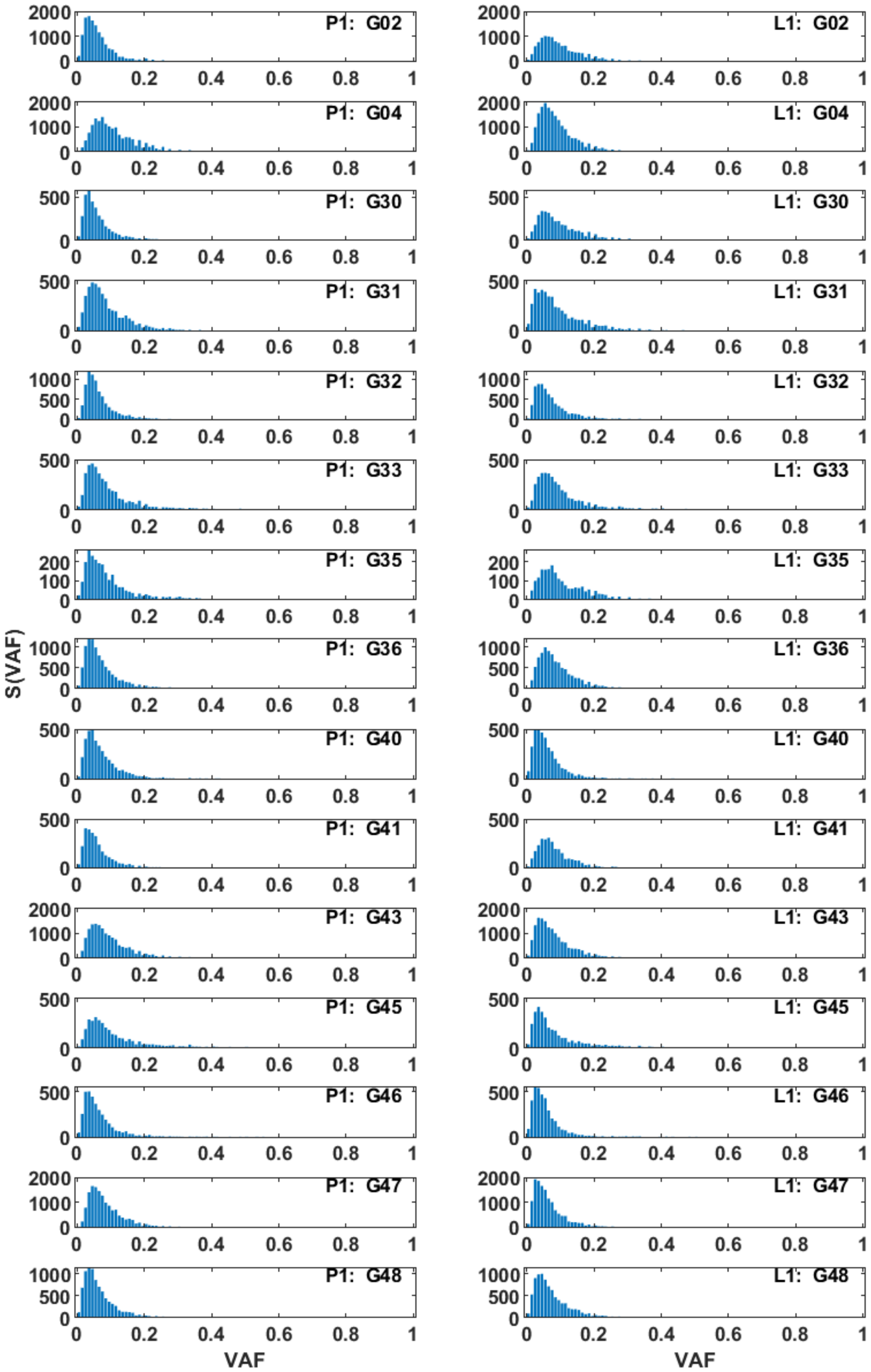

Figure S9: SFS based on VAF for all samples (unfiltered) for primary tumor (P1, left column) and lymph node metastasis (L1, right column).

bioRxiv preprint doi: https://doi.org/10.1101/2025.10.04.680485; this version posted October 5, 2025. The copyright holder for this preprint (which was not certified by peer review) is the author/funder, who has granted bioRxiv a license to display the preprint in perpetuity. It is made available under aCC-BY 4.0 International license.

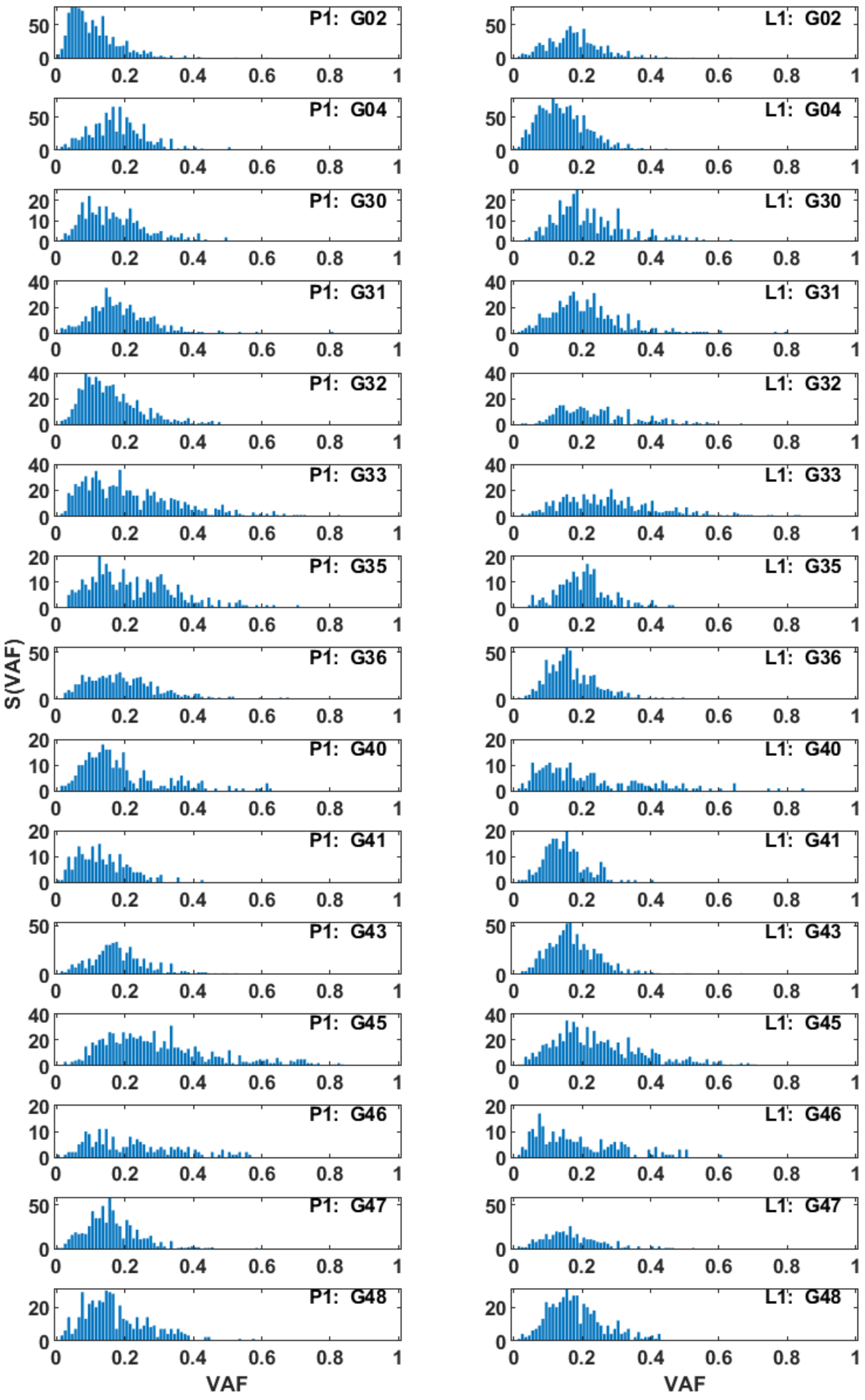

Figure S10: SFS based on VAF for all samples (filtered with DEEPOMICS with FFPE score threshold= 0.25) for primary tumor (P1, left column) and lymph node metastasis (L1, right column).

bioRxiv preprint doi: https://doi.org/10.1101/2025.10.04.680485; this version posted October 5, 2025. The copyright holder for this preprint (which was not certified by peer review) is the author/funder, who has granted bioRxiv a license to display the preprint in perpetuity. It is made available under aCC-BY 4.0 International license.

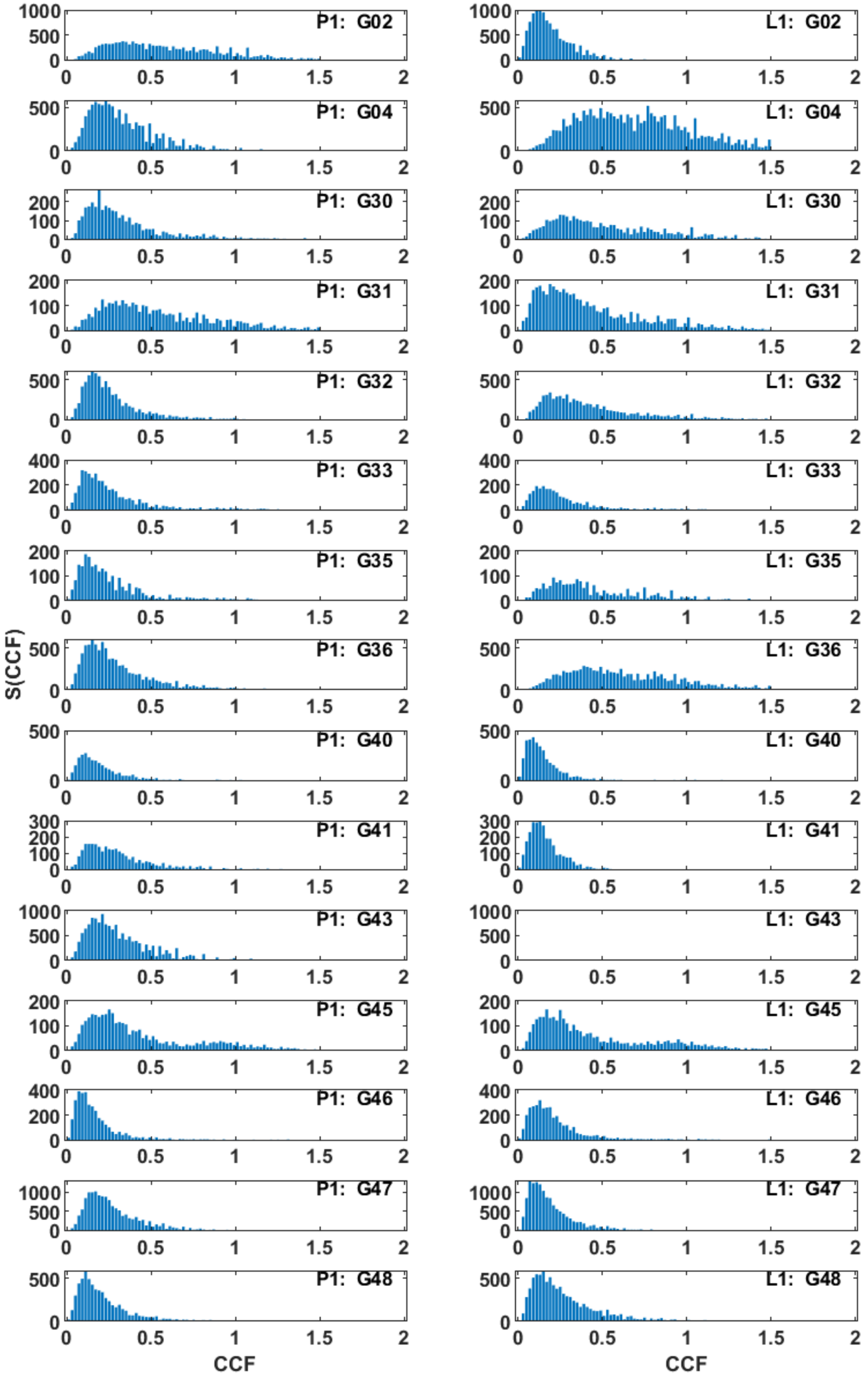

Figure S11: SFS based on CCF for all samples (unfiltered) for primary tumor (P1, left column) and lymph node metastasis (L1, right column).

bioRxiv preprint doi: https://doi.org/10.1101/2025.10.04.680485; this version posted October 5, 2025. The copyright holder for this preprint (which was not certified by peer review) is the author/funder, who has granted bioRxiv a license to display the preprint in perpetuity. It is made available under aCC-BY 4.0 International license.

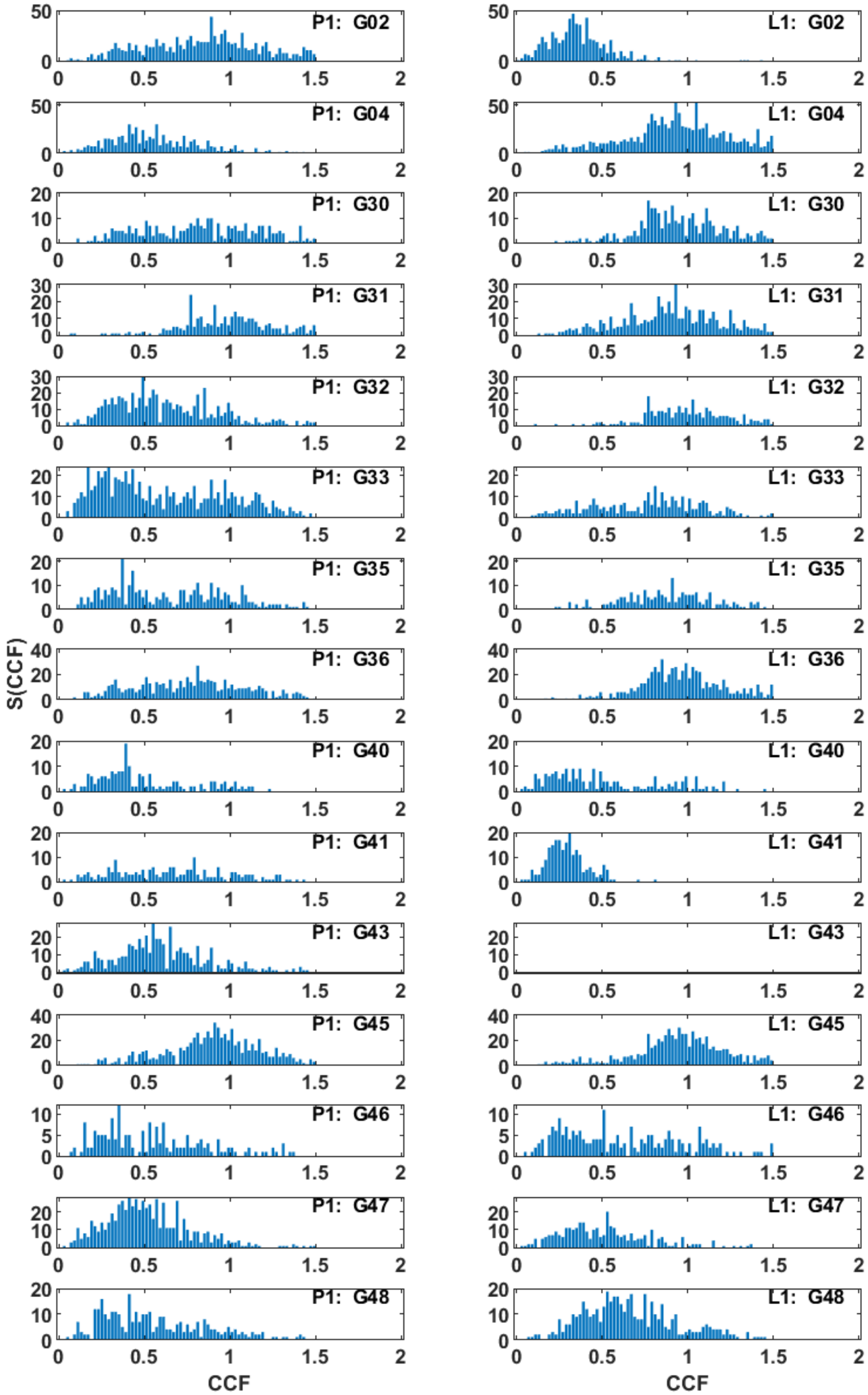

Figure S12: SFS based on CCF for all samples (filtered with DEEPOMICS with FFPE score equal to 0.25) for primary tumor (P1, left column) and lymph node metastasis (L1, right column).

bioRxiv preprint doi: https://doi.org/10.1101/2025.10.04.680485; this version posted October 5, 2025. The copyright holder for this preprint (which was not certified by peer review) is the author/funder, who has granted bioRxiv a license to display the preprint in perpetuity. It is made available under aCC-BY 4.0 International license.

B.3 Trade-off between purity and ploidy estimates

For each purity-ploidy pair ASCAT [42] rounds the inferred tumor copy numbers to integers and computes how well these integers determine the observed LogR and BAF [39]. Different integer assignments yield locally good fits, producing disjoint curved bands (valleys) of low error in which the segments can be explained by the same integer copy number combination (Fig. S13). In some cases, the result obtained might be ambiguous due to trade-off between purity and ploidy estimates, which results in multiple regions with low error.

Copy number plots (Fig. S14) show pointwise SNP data across the genome with two rows: the per-locus LogR ($\log_2$ total intensity ratio) and the BAF (B-allele fraction). LogR reports changes in total DNA amount while BAF reports allelic imbalance — ASCAT fits both jointly (under a tumor+normal mixture and integer tumor copy numbers) to infer purity, ploidy and allele-specific copy number.

bioRxiv preprint doi: https://doi.org/10.1101/2025.10.04.680485; this version posted October 5, 2025. The copyright holder for this preprint (which was not certified by peer review) is the author/funder, who has granted bioRxiv a license to display the preprint in perpetuity. It is made available under aCC-BY 4.0 International license.

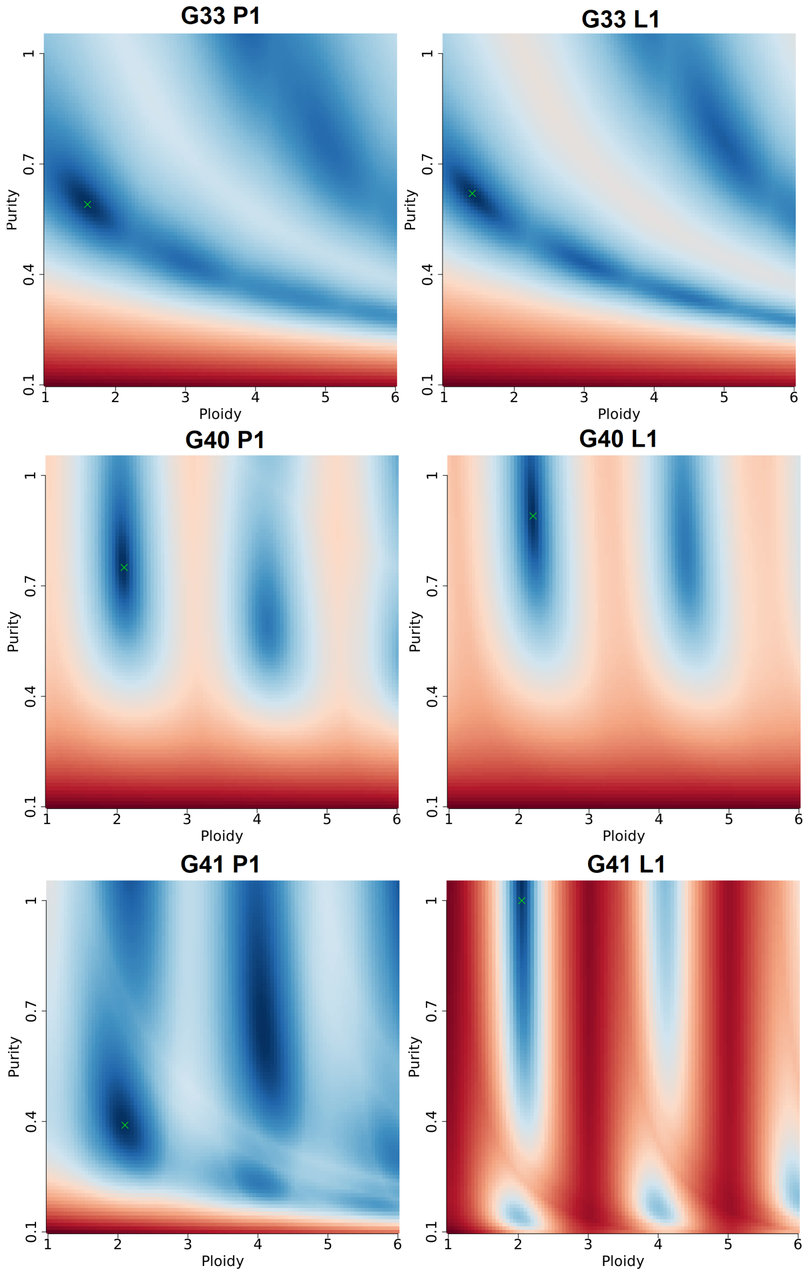

Figure S13: Ploidy-purity plots for patients G33, G40 and G41 for primary tumor (P1, left column) and lymph node metastasis (L1, right column). Sunrise plots represent a fit-score surface over a grid of candidate purity (y-axis) and ploidy (x-axis) values. Color encodes goodness of fit: dark blue regions represent good fit (low error), red regions correspond to worse fit. The green crosses mark the ASCAT-selected solutions (the ploidy-purity pairs chosen for downstream integer copy-number calling).

bioRxiv preprint doi: https://doi.org/10.1101/2025.10.04.680485; this version posted October 5, 2025. The copyright holder for this preprint (which was not certified by peer review) is the author/funder, who has granted bioRxiv a license to display the preprint in perpetuity. It is made available under aCC-BY 4.0 International license.

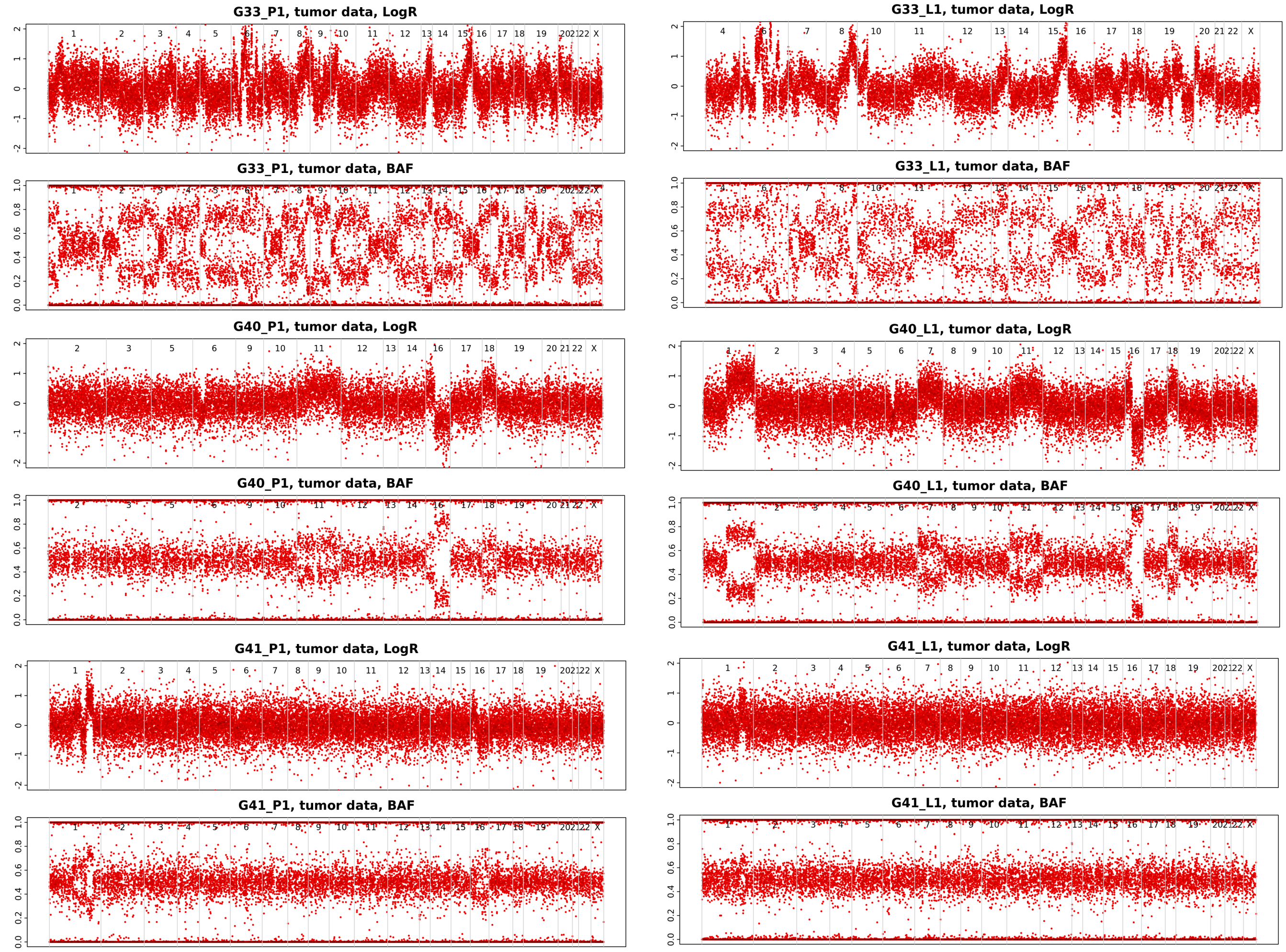

Figure S14: Copy number plots after GC and replication-timing (RT) correction for patients G33, G40 and G41 for primary tumor (P1, left column) and lymph node metastasis (L1, right column) show pointwise SNP data across the genome in the form of the per-locus LogR (top panel) and the BAF (bottom panel). Vertical separators mark chromosomes.
